# The Feasibility of Using Guided Self-Help in Anorexia Nervosa: An Analysis of Drop-Out From the Study Protocol and Intervention Adherence

**DOI:** 10.3389/fpsyg.2020.00707

**Published:** 2020-04-16

**Authors:** Valentina Cardi, Gaia Albano, Laura Salerno, Gianluca Lo Coco, Suman Ambwani, Ulrike Schmidt, Pamela Macdonald, Janet Treasure

**Affiliations:** ^1^Section of Eating Disorders, Department of Psychological Medicine, Institute of Psychiatry, Psychology and Neuroscience, King’s College London, London, United Kingdom; ^2^Department of Psychology, Educational Sciences and Human Movement, University of Palermo, Palermo, Italy; ^3^Dickinson College, Carlisle, PA, United States

**Keywords:** anorexia nervosa, drop-out, intervention, motivation, online, trial

## Abstract

The implementation of online technologies to promote wellbeing is increasingly becoming a worldwide priority. This study includes secondary analyses of data and examined drop-out rates in an online guided self-help intervention for patients with anorexia nervosa. Specifically, rates of drop-out at end of treatment (i.e., 6 weeks assessment), as well as intervention adherence (minimum of four of six online guided sessions) and differences between completers and drop-outs were examined. Motivation to change and associated patient variables were assessed as predictors of drop-out using structural equation modeling. Ninety-nine patients were randomized to the intervention arm of the trial. Data were available for 82 individuals, 67 of whom completed the 6 weeks assessment and attended a minimum of four online sessions. No significant differences were found between completers and drop-outs at baseline. At the end of the first week of participation, drop-outs from the 6 weeks assessment or the intervention reported less satisfaction with their work with the mentor delivering online guidance. Greater confidence in own ability to change and higher controlled motivation (willingness to change due to pressure from others) predicted lower drop-out rates from the 6 weeks assessment. Stronger alliance with the therapist at the treatment center and lower psychological distress were associated with greater autonomous motivation (self-directed motivation) and importance and ability to change. Data demonstrate that a novel online guided self-help intervention for patients with anorexia nervosa is feasible. Early satisfaction with the program and external pressure to change have a protective role against drop-out rates.

**Clinical Trial Registration:** www.ClinicalTrials.gov, identifier NCT02336841.

## Introduction

The World Health Organization (WHO) has established the use of online technologies to support wellbeing (eHealth) as a priority (World Health Organization, 2016). This challenge has been embraced by mental health professionals and researchers, as demonstrated by a large increase in the utilization of technological aids in the prevention and treatment of mental health issues (Zhang and Ho, 2015). One of the main advantages of developing and implementing online mental health programmes is that they can be more easily disseminated to, and received by patients, compared to standard face-to-face therapies. This is particularly relevant for mental illnesses that are difficult to identify, for which access to specialized services is challenging and that are marked by high levels of stigmatization and shame. At the same time, concerns have been raised regarding the high drop-out rates from online interventions (on average 31%) among people with psychological disorders (Melville et al., 2010) and recent studies have highlighted the need for more research on patient individual factors associated with drop-out (Fernández-Álvarez et al., 2017). The aim of this paper is to examine dropout from a novel online guided self-help intervention in anorexia nervosa.

Patients with eating disorders are difficult to identify and treat, despite the burden that the illness poses on the individual, their families and the society (Aardoom et al., 2016). Only a subgroup of individuals receives appropriate treatment (Hart et al., 2011), whilst others struggle with barriers such as poor availability of specialized services and high levels of shame and fear of criticism related to the illness (Cachelin and Striegel-Moore, 2006; Becker et al., 2010). In recent years, there has been a large increase in the use of computerized interventions for patients with eating disorders, especially for prevention and to treat symptoms of loss of control over-eating and purging using cognitive-behavioral principles and techniques (Aardoom et al., 2013; Schlegl et al., 2015). Most of these interventions include self-help materials and different forms of guidance delivered by health professionals or lay people and are overall associated with reduced eating disorder psychopathology (medium effect size) and binge abstinence (small effect size) (Traviss-Turner et al., 2017). However, drop-out rates from manualized self-help interventions for eating disorders vary greatly across studies (ranging between 1 and 88%; Beintner et al., 2014), and intervention- and person-related variables associated with early drop-out from study protocols and interventions are largely unknown (e.g., Barakat et al., 2019).

Data on the efficacy and acceptability of online treatments are particularly scarce in anorexia nervosa. This might be justified by cautiousness and concerns regarding the use of non-traditional forms of therapy (e.g., regular and intensive face-to-face contact with a mental health professional) with individuals at risk of medical complications (Wilson and Zandberg, 2012). However, more recent findings from a systematic review and meta-analysis on task-sharing interventions in anorexia nervosa (Albano et al., 2019) suggest that guided self-help in this condition is associated with lower drop-out rates from the study protocol than a comparison condition (either waiting list or inpatient/outpatient treatment protocols). Based on this evidence, as well as the high rates of patients who do not complete or relapse from treatment and their strong ambivalence toward change (Schmidt and Treasure, 2006; Fassino et al., 2009; DeJong et al., 2012) we suggest that the use of online guided self-help to complement standard care in anorexia nervosa is worth exploring.

We developed a 6 weeks online guided self-help intervention for patients with anorexia nervosa (*Recovery*MANTRA) and compared the efficacy of adding this intervention to Treatment As Usual (TAU; standard care consisting of medical monitoring and psychological support) against TAU alone in a randomized controlled trial (i.e., SHARED) of patients with anorexia nervosa assessed for outpatient treatment (Cardi et al., 2015). Findings indicated that patients receiving *Recovery*MANTRA in addition to TAU reported higher confidence in own ability to change (*p* = 0.02, small effect size), greater alliance with the therapist at the outpatient service (*p* = 0.005, small to medium effect size) and trend-level greater reductions in anxiety (*p* = 0.06, small effect size) at 6 weeks, compared to a control group (Cardi et al., 2019). *Recovery*MANTRA challenges positive beliefs about the illness and other maintaining factors, including cognitive rigidity, emotion regulation difficulties, isolation and food restriction (Schmidt and Treasure, 2006; Treasure and Schmidt, 2013). It focuses on the use of behavior change techniques and weekly online support from mentors (i.e., recovered individuals, carers of people with lifetime eating disorders) and graduate psychology students trained in motivation interviewing (Cardi et al., 2015). The greatest emphasis of *Recovery*MANTRA is on empowering individuals by increasing their motivation and confidence to change (Cardi et al., 2015). This is consistent with the assumptions of self-determination theory that underpins the intervention and also with the evidence that a patient’s motivation to change predicts outcome and drop-out in eating disorders (Vall and Wade, 2015; Thaler et al., 2016). Patient autonomous motivation to change before treatment (i.e., motivation to change due to a patient’s intrinsic motivation), in particular, appears related to lower levels of eating disorder symptoms at the end of treatment (Mansour et al., 2012; Thaler et al., 2016) or to faster improvement in these symptoms (Carter and Kelly, 2015). On the other hand, controlled motivation (i.e., motivation to change due to pressure from others or the desire to avoid negative feelings, such as shame and guilt) has not been found to predict treatment outcomes (Mansour et al., 2012; Carter and Kelly, 2015; Thaler et al., 2016).

This study conducted secondary analyses of data from the SHARED trial (as published in Cardi et al., 2015, 2019) and examined drop-out rates (i.e., non-completion of end-of-intervention assessment measures) and intervention adherence rates (adherence defined as attendance of a minimum of four of six sessions) to establish the acceptability of delivering *Recovery*MANTRA to patients. The drop-out and completer groups were compared in terms of baseline socio-demographic and clinical variables and eating behaviors, usage of the self-help materials and perceived quality of the relationship with the online mentor at the end of the first week of project participation. Baseline motivation to change among patients (i.e., autonomous motivation, controlled motivation, importance to change and confidence in own ability to change) and related patient variables were considered to predict drop-out from the 6 weeks assessment and drop-out from the intervention.

Based on a number of studies available in the literature on the use of technology-based interventions in eating disorders (Schlegl et al., 2015), no differences in clinical (i.e., illness severity) or demographic (i.e., age, years of education) variables between those who did and did not drop-out were expected at baseline. However, it was hypothesized that there would be differences between groups in terms of perceived quality of the relationship with the mentor within the first week of receiving *Recovery*MANTRA (for a review on the importance of considering process measures earlier on when delivering technology-based interventions; see Kelders et al., 2012). In particular, it was expected for completers to report greater satisfaction with the mentor allocated to them and their work together. Based on past findings in the eating disorder literature (Mansour et al., 2012; Carter and Kelly, 2015; Thaler et al., 2016), it was also predicted that higher levels of autonomous motivation to change and higher levels of importance and confidence in one’s own ability to change would be associated with lower rates of drop-out from the end-of-intervention assessment and from *Recovery*MANTRA.

## Materials and Methods

### Participants

This longitudinal study was part of a multi-center, two-armed trial comparing the effects of treatment as usual (TAU) complemented by guided self-help (*Recovery*MANTRA) to the effects of TAU alone on clinical outcomes of patients with anorexia nervosa assessed for outpatient treatment (Cardi et al., 2015, 2019). The purpose of this study was to investigate drop-out rates from completing the assessment measures at the end of the intervention (i.e., drop-out from the assessment) as well as drop-out from *Recovery*MANTRA (i.e., drop-out from the intervention defined as attendance of less than four out of six online guided sessions) in the group of individuals randomized to receive *Recovery*MANTRA in addition to TAU. This group was composed of 99 individuals, aged 16 or over and with a diagnosis of anorexia nervosa or atypical/partial anorexia nervosa according to the Diagnostic and Statistical Manual of Mental Disorders, 5th Edition (American Psychiatric Association, 2013; definition of atypical anorexia nervosa as follows: fulfillment of all diagnostic criteria, except the weight criterion or amenorrhea or fat phobia; definition of partial anorexia nervosa, as follows: having features of the illness, but missing at least two of the four diagnostic criteria, Thomas et al., 2009). Participants were recruited between April 2015 and December 2016 from 22 eating disorder outpatient services across the United Kingdom. The investigation was carried out in accordance with the latest version of the Declaration of Helsinki and the study design was reviewed by an appropriate ethical committee (Research Ethics Committee of London-Brent, project reference number: 14-LO-1347). Informed consent of the participants was obtained after the nature of the procedures had been fully explained. Exclusion criteria were: (a) life-threatening anorexia nervosa as defined in the NICE guidelines, (b) insufficient knowledge of English, and (c) severe mental or physical illness needing treatment in its own right (e.g., psychosis or diabetes mellitus). Due to missing data on key baseline variables, 17 subjects were excluded from the analyses. The final sample included 82 subjects and their clinical and sociodemographic characteristics are shown in Table 1.

**TABLE 1:** Participants’ demographics and clinical variables.

		Drop-out from the assessment				Drop-out from the intervention			
			
	Total group	Completers	Drop-outs	Test and	Cohen’s	Completers	Drop-outs	Test and	Cohen’s
	(*n* = 82)	(*n* = 67)	(*n* = 15)	*p*-values	d ES	(*n* = 70)	(*n* = 12)	*p*-values	d ES
	
				Completer vs.				Completer vs.	
				Drop-out				Drop-out	
	Mean (*SD*)	Mean (*SD*)	Mean (*SD*)	groups		Mean (*SD*)	Mean (*SD*)	groups	
**BASELINE VARIABLES**									
Age	26.57 (8.29)	27.03 (8.86)	24.53 (4.67)	*t*(80) = 1.05 *p* = 0.294	0.35	26.81 (8.73)	25.17 (4.99)	*t*(80) = 0.63 *p* = 0.528	0.23
Years of education	15.78 (2.59)	15.87 (2.54)	15.33 (2.87)	*t*(72) = 0.66 *p* = 0.514	0.20	15.85 (2.50)	15.33 (3.28)	*t*(72) = 0.55 *p* = 0.581	0.18
Body mass index	16.09 (1.41)	16.06 (1.41)	16.24 (1.43)	*t*(80) = −0.45 *p* = 0.652	0.13	16.06 (1.42)	16.24 (1.39)	*t*(80) = −0.40 *p* = 0.690	0.13
Duration of illness	6.75 (7.80)	7.22 (8.33)	4.67 (4.35)	*t*(80) = 1.15 *p* = 0.255	0.38	7.04 (8.20)	5.08 (4.75)	*t*(80) = 0.80 *p* = 0.426	0.29
Eating Disorder Examination Questionnaire	4.01 (1.14)	3.91 (1.13)	4.43 (1.13)	*t*(80) = −1.61 *p* = 0.112	0.46	3.92 (1.10)	4.53 (1.26)	*t*(80) = −1.75 *p* = 0.084	0.52
Depression Anxiety and Stress Scales	59.71 (23.49)	58.15 (21.99)	66.67 (29.13)	*t*(80) = −1.27 *p* = 0.206	0.33	57.89 (21.78)	70.33 (30.75)	*t*(80) = −1.72 *p* = 0.090	0.47
Work and Social Adjustment Scale	19.91 (7.86)	19.85 (7.60)	20.20 (9.21)	*t*(80) = −0.15 *p* = 0.877	0.04	19.90 (7.58)	20.00 (9.70)	*t*(80) = −0.04 *p* = 0.968	0.01
Importance to change	7.85 (2.19)	7.76 (2.22)	8.27 (2.09)	*t*(80) = −0.81 *p* = 0.422	0.24	7.81 (2.20)	8.08 (2.19)	*t*(80) = −0.39 *p* = 0.697	0.12
Confidence in own ability to change	5.19 (2.34)	5.32 (2.31)	4.60 (2.47)	*t*(80) = 1.09 *p* = 0.279	0.30	5.34 (2.29)	4.33 (2.57)	*t*(80) = 1.39 *p* = 0.169	0.41
Autonomous Motivation	4.84 (0.98)	4.83 (1.02)	4.86 (0.81)	*t*(80) = −0.08 *p* = 0.937	0.03	4.86 (1.01)	4.72 (0.82)	*t*(80) = 0.44 *p* = 0.662	0.15
Controlled Motivation	4.63 (0.91)	4.69 (0.93)	4.36 (0.78)	*t*(80) = 1.29 *p* = 0.202	0.38	4.63 (0.96)	4.60 (0.61)	*t*(80) = 0.13 *p* = 0.900	0.04
Alliance with therapist	4.84 (1.30)	4.92 (1.26)	4.49 (1.46)	*t*(80) = 1.14 *p* = 0.256	0.31	4.95 (1.24)	4.17 (1.45)	*t*(80) = 1.98 *p* = 0.051	0.58
Cognitive and behavioral flexibility	3.51 (1.06)	3.50 (0.97)	3.53 (1.43)	*t*(80) = −0.11 *p* = 0.913	0.02	3.53 (1.00)	3.37 (1.38)	*t*(80) = 0.46 *p* = 0.645	0.13
**VARIABLES AT WEEK 1**									
Confidence in own ability to change week 1	2.53 (1.02)	2.62 (1.01)	2.00 (0.89)	*t*(76) = 1.88 *p* = 0.064	0.65	2.59 (1.01)	2.00 (0.93)	*t*(76) = 1.56 *p* = 0.123	0.61
Hope week 1	2.53 (1.03)	2.60 (1.00)	2.00 (1.09)	*t*(76) = 1.81 *p* = 0.074	0.57	2.57 (1.00)	2.00 (1.19)	*t*(76) = 1.50 *p* = 0.138	0.52
Restriction week 1	0.75 (1.05)	0.72 (1.01)	0.92 (1.26)	*t*(78) = −0.65 *p* = 0.519	0.17	0.71 (1.01)	1.00 (1.33)	*t*(78) = −0.65 *p* = 0.529	0.25
Purging week 1	0.30 (0.75)	0.19 (0.63)	0.85 (1.07)	*t*(78) = −2.13 *p* = 0.052	0.61	0.21 (0.66)	0.90 (1.10)	*t*(78) = −1.92 *p* = 0.084	0.76
Use of self−help materials week 1	1.68 (0.47)	1.65 (0.48)	1.80 (0.42)	*t*(60) = −0.98 *p* = 0.344	0.33	1.67 (0.47)	1.71 (0.49)	*t*(60) = −0.22 *p* = 0.828	0.08
Comfortable working with mentor week 1	5.04 (1.68)	5.19 (1.61)	4.00 (1.87)	*t*(70) = 2.03 *p* = 0.046	0.68	5.17 (1.62)	3.67 (1.86)	*t*(70) = 2.14 *p* = 0.036	0.86
Agreed goals with mentor week 1	4.82 (1.74)	5.06 (1.60)	3.30 (1.89)	*t*(71) = 3.15 *p* = 0.002	1.00	4.94 (1.69)	3.71 (1.98)	*t*(71) = 1.80 *p* = 0.077	0.67

*Demographic and clinical variables are expressed as means and standard deviations (SDs). Means and SDs are presented for the entire sample, as well as for completers and drop-outs, separately.*

### Measures

Participants completed a baseline assessment consisting of the following measures:

*Demographic and clinical survey*, to collect information on age, gender, ethnicity, years of education, employment and social status, duration of illness, time of illness onset, diagnosis and first treatment received, previous hospital admissions, psychiatric comorbidity and medication and self-reported body mass index (BMI).

*Autonomous and Controlled Motivations for Treatment Questionnaire* (ACMTQ; Zuroff et al., 2007), a 12-item self-report questionnaire which consists of two six-item subscales assessing autonomous motivation and controlled motivation for treatment. Participants are asked to rate the extent to which they agree with each statement using a seven-point rating scale. The ACMTQ showed good/acceptable internal consistency in this study (Cronbach’s α values: 0.89 and 0.71 for autonomous and controlled motivation subscales, respectively).

*Importance and confidence in own ability to change* were assessed using two self-developed single-items Likert scales ranging from 1 (“not important at all”/“not confident at all”) to 10 (“extremely important”/“extremely confident in my ability to change”). This questionnaire is available in Supplementary Material.

*Eating Disorder Examination Questionnaire* (EDE-Q; Fairburn and Beglin, 1994), a 36-item self-report measure of eating disorder symptoms. The EDE-Q has been widely validated in clinical and non-clinical groups (Mond et al., 2004) and shows good reliability and validity. Items are rated on a six-point Likert scale, where higher scores indicate a greater level of eating pathology. For the purpose on the present study, only the total score was used (Cronbach’s α:0.92).

*Depression, Anxiety and Stress Scales* (DASS-21; Lovibond and Lovibond, 1995) is a 21-item self-report measure of patients’ psychological distress over the past 7 days. Items are scored on a four-point Likert scale. It includes three subscales (i.e., anxiety, depression, and stress), but only the total score was considered in this study (Cronbach’s α:0.91).

*Work and Social Adjustment Scale* (WSAS; Mundt et al., 2002), a five-item self-report scale designed to assess patients’ perceptions of impairment in everyday functioning resulting from a given problem. The scale evaluates functioning in the following domains: work, home management, social and private leisure activities, and close relationships. Scores for each item range from 0 to 8 and higher scores reflect more severe functional impairment. The WSAS demonstrated acceptable internal consistency in this study (Cronbach’s α:0.73).

*Alliance with therapist* delivering TAU at the outpatient treatment centre was evaluated using five self-developed visual analogs scales [ranging from 0 (never) to 7 (always)] assessing patients’ feelings that the therapist understood them, could be trusted, and that they worked toward mutually agreed and relevant goals. A mean score of the five scales was calculated to reflect overall alliance and used in this study (Cronbach’s α: 0.92). This questionnaire is available in Supplementary Material.

*Cognitive and behavioral flexibility* were assessed using four self-developed visual analog scales (ranging from 0 – never, to 7 – always) measuring the patient’s attention to details and use of rigid behaviors. This questionnaire is available in Supplementary Material.

These measures, except for the demographic and clinical survey, were repeated at 6 weeks. Additionally, patients completed daily assessments of importance and confidence in their ability to change and hope (all measured using visual analogue scales ranging from 1 “not at all” to 5 “extremely”). They also completed weekly measures of frequency of eating disorder behaviors (restriction, purging, over-exercising, on a Likert scale ranging from 0: “0 days,” to 3: “6–7 days”), usage of self-help materials (workbook and video-clips, on a Likert scale ranging from 1 “0 days” to 5 “6–7 days”) and alliance with their mentor for the online sessions (i.e., ease of working with the mentor and degree to which they both agreed on the goals for the sessions, measured on a Likert scale ranging from 1 “never” to 7 “always”).

### Procedure

Participants were recruited within a month from their first assessment session at the outpatient service. They completed the online baseline measures listed above on the study’s website and were then randomized, based on treatment centre and illness severity (i.e., Body Mass Index < 16 or ≥ 16 kg/m^2^) to one of two study conditions: *Recovery*MANTRA plus TAU, or TAU alone. Participants in both groups completed an online assessment at 6 weeks and at 6- and 12-month follow-up (Cardi et al., 2015).

### *Recovery*MANTRA and Treatment as Usual (TAU)

Participants allocated to the *Recovery*MANTRA + TAU group, had access to online self-help materials (workbook and video-clips) and weekly 1 h, individual, synchronous text-based chat sessions with a peer mentor or mentor. The aim of the guidance was to help participants to understand and familiarize with the contents provided by the self-help materials effectively and purposefully, in order to supplement their TAU. Peer mentors and mentors were respectively individuals recovered from an eating disorder and students and were trained in the use motivational interviewing strategies.

The exact content of TAU varied between the recruitment centers, but overall consisted of psychoeducation, individual or group psychotherapy, nutritional support, and medical monitoring.

### Statistical Analyses

**FIGURE 1:**
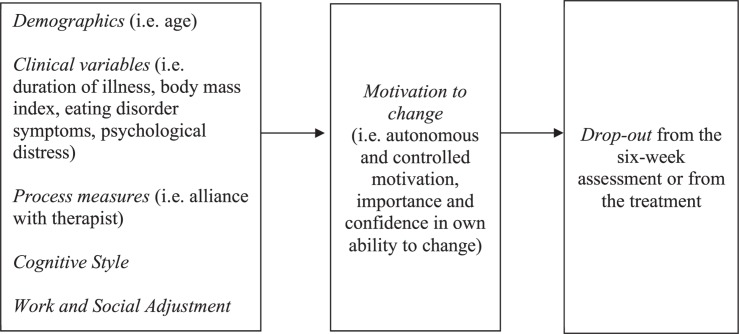
Theoretical model. This figure describes hypothesized relationships between the variables investigated.

Demographic and clinical variables were described using means and standard deviations or percentages. The baseline and week 1 differences between groups were investigated using independent samples *t*-tests. Bivariate (Pearson) correlations coefficients between variables were computed. A structural equation model (SEM) was tested to analyze the relationships between baseline patient variables, motivation to change and drop-out from end of 6 weeks assessment or the intervention. SEM consists of a set of multivariate techniques that are confirmatory rather than exploratory in testing model fit (Byrne, 2011). It allows simultaneous and comprehensive estimation of the hypothesized relations among multiple independent and dependent variables in the model using the estimated covariance matrix generated on the basis of the observed covariance matrix of the measured variables. Model testing was performed using Mplus 6.0 (Muthén and Muthén, 1998–2012). A theoretical representation of the tested model is shown in Figure 1. Skewness and kurtosis were assessed and the Weighted Least Squares Mean and Variance adjusted (WLSMV) estimator was used as the method of parameter estimation. The following indices were considered to evaluate the overall model goodness fit: χ^2^-test statistics (χ^2^/df ratios < 3 indicate models with reasonable fit, Schermelleh-Engel et al., 2003), the comparative fit index (CFI, with values between 0.80 and 0.89 indicating adequate but marginal fit and values of ≥0.95 indicating better fit, Hu and Bentler, 1999) and the root-mean-square error of approximation (RMSEA, with values of ≤0.05 indicating close fit, and < 0.08 indicating reasonable fit) (Hoyle and Panther, 1995; MacCallum et al., 1996).

## Results

### Demographic and Clinical Characteristics

Most participants were female (80/82) and from a white ethnic background (75/82). The mean age was 26.57 years (*SD* = 8.29). Almost half of the sample (41/82) was employed (part-time or full-time) and were not in a relationship (43/82). The mean body mass index (BMI) was 16.09 kg/m^2^ (*SD* = 1.41). On average, patients had been ill for 7 years (*SD* = 7.80). A subgroup reported psychiatric comorbidity (*n* = 19), a previous hospital admission (*n* = 20) or the use of psychiatric medication (*n* = 34). Twenty-three participants (28%) reported purging symptoms. Demographic and clinical variables are described in Table 1.

Pearson correlations coefficients are shown in Table 2. At baseline, greater alliance with the therapist delivering TAU and lower psychological distress were related to higher autonomous motivation (*p* < 0.01) and importance and confidence in own ability to change (*p* < 0.01). Patients with lower BMI also reported higher importance to change (*p* < 0.05). However, this finding needs to be interpreted cautiously, considering that BMI was self-reported (as opposed to being measured by a clinician).

**TABLE 2:** Pearson correlation coefficients of the study’s variables.

	1	2	3	4	5	6	7	8	9	10	11	12	13
1. Age	–												
2. Alliance with therapist	0.237*	–											
3. Work and Social Adjustment Scale	–0.006	–0.129	–										
4. Depression Anxiety and Stress Scales	–0.173	−0.223*	0.537**	–									
5. Cognitive style	0.083	0.174	–0.195	–0.146	–								
6. Duration of illness	0.623**	0.216	0.091	–0.010	0.080	–							
7. Body mass index	–0.142	–0.039	–0.130	–0.018	0.038	–0.141	–						
8. Eating Disorder Examination Questionnaire	–0.070	–0.175	0.348**	0.652**	−0.229*	0.125	0.039	–					
9. Autonomous motivation	–0.066	0.398**	–0.003	−0.304**	0.054	–0.090	–0.094	–0.150	–				
10. Controlled motivation	0.018	0.198	0.165	0.007	–0.058	–0.007	–0.192	–0.016	0.372**	–			
11. Importance to change	0.028	0.343**	0.080	−0.292**	0.054	0.067	−0.271*	–0.136	0.577**	0.123	–		
12. Confidence in own ability to change	–0.012	0.395**	−0.275*	−0.501**	0.164	–0.108	0.020	−0.290**	0.515**	–0.026	0.460**	–	
13. Dropped-out from the assessment	–0.117	–0.127	0.017	0.141	0.012	–0.127	0.051	0.177	0.009	–0.142	0.090		–
14. Dropped-out from the intervention	–0.071	–0.216	0.005	0.188	–0.052	–0.089	0.045	0.192	–0.049	–0.014	0.044	–0.153	0.875**

*p < 0.05; **p < *0.01.*

### Completion of 6-Week Assessment and Guided Sessions

**FIGURE 2:**
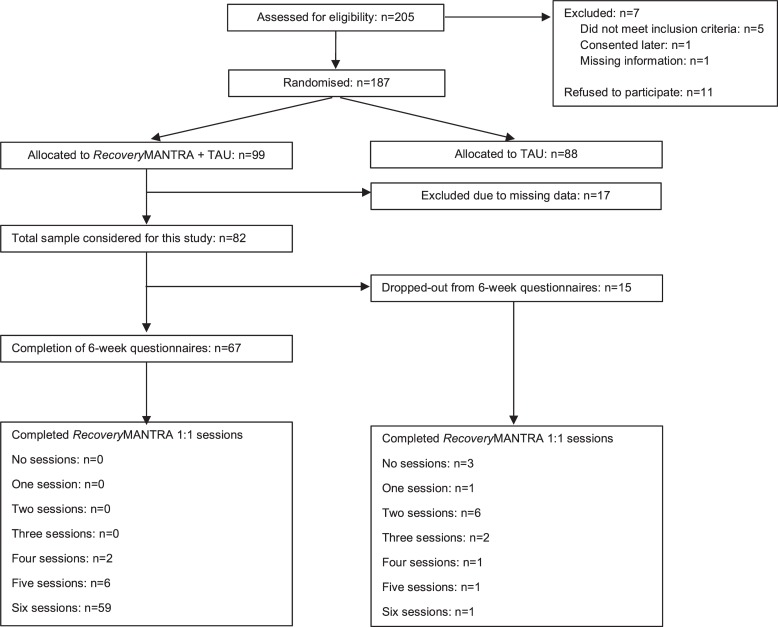
Study flow-chart. This figure describes the process of assessing, randomizing and assessing participants and includes number of participants who completed the 6 weeks assessments and the *Recovery*MANTRA intervention sessions.

Rates of completion of the online assessments and attendance of the six guided sessions are shown in Figure 2. Sixty-seven participants completed the 6 weeks assessment, of whom all attended at least four guided sessions (*n* = 2 patients attended four sessions; *n* = 6 attended five sessions; *n* = 59 attended six sessions). Fifteen participants did not complete the 6 weeks questionnaires. Among those, 12 completed less than four sessions (*n* = 3 patients attended no sessions, *n* = 1 completed one session, *n* = 6 completed two sessions, *n* = 2 completed three sessions, *n* = 1 completed four sessions, *n* = 1 completed five sessions, *n* = 1 completed six sessions).

Forty-nine participants (59.8%) received online support from graduate psychology students and 33 participants (40.2%) received online support from people with lived experience of eating disorders (recovered individuals or carers of people with lifetime eating disorders). The type of mentor did not impact on levels of drop-out.

### Baseline Differences Between Completers and Drop-Outs

Overall, there were no statistically significant differences in terms of demographic and clinical characteristics between the group of individuals who completed the 6 weeks assessment or the intervention and those who did not (Table 1). There was a trend (*p* = 0.05, medium effect size) for those who did not complete the intervention to report lower alliance with their therapist at the outpatient clinic (Table 1).

### Differences Between Groups at the End of the First Week of Participation in the Program

Patients who did not complete the end-of-intervention measures felt less comfortable working with their mentors (*p* < 0.05, medium effect size) and showed lower levels of agreement with them on the goals for the sessions (*p* < 0.01, large effect size) at the end of their first week of participation. There were also trends for participants who dropped out to report more episodes of purging (*p* = 0.05, medium effect size) and to have lower confidence in their ability to change (*p* = 0.06, medium effect size).

Participants who completed less than four online sessions felt less comfortable working with their mentor at the end of the first week of their participation in the program (*p* < 0.05, large effect size).

### Structural Equation Modeling

**FIGURE 3:**
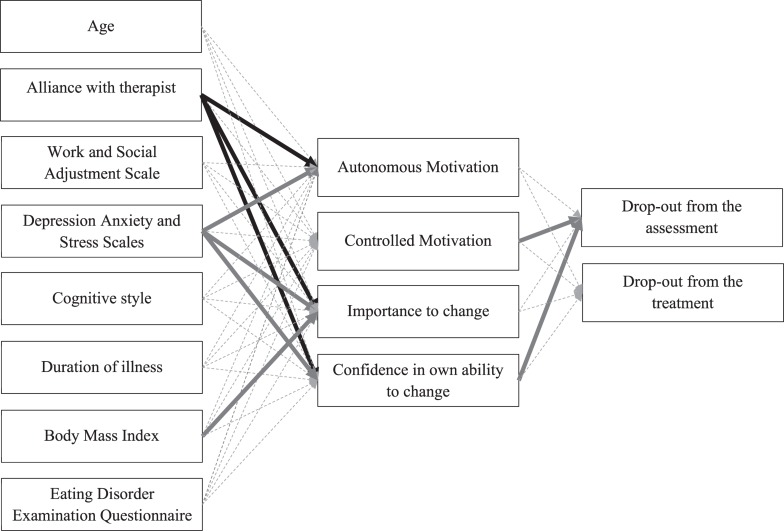
Structural equation model. This model describes the relationships between patient demographic and clinical variables, self-reported motivation and drop-out from the 6 weeks assessments and *Recovery*MANTRA intervention. Errors are omitted from the diagram. Significant positive parameters are represented by black solid lines. Significant negative parameters are represented by gray solid lines. Non-significant parameters are represented by gray dashed lines. For clarity, correlations between variables are omitted from the diagram: autonomus motivation is not significantly correlated to controlled motivation (*p* < 0.001), importance to change (*p* < 0.01) and confidence in ability to change (*p* < 0.01); moreover, importance to change is correlated to confidence in ability to change (*p* < 0.01) and drop-out from the assessment is correlated to drop-out from the treatment (*p* < 0.001).

Figure 3 shows the hypothesized model of the relationships among age, clinical impairment (eating disorder symptoms, body mass index, duration of illness, psychological distress), cognitive style, alliance with the therapist at the outpatient center, social and work adjustment, autonomous and controlled motivation for treatment, importance and confidence in own ability to change at baseline and drop-out from the assessment and from the intervention. The model showed a good fit to the data considering the following parameters: χ^2^ = 15.573, *df* = 18, χ^2^/*df* = 0.86, CFI = 1.000, RMSEA = 0.000, RMSEA 90% CI = 0.000–0.084. The standardized parameter estimates in Table 3 indicated that the alliance with the therapist delivering TAU at the outpatient service at baseline was associated with all aspects of patient motivation to change (i.e., autonomous motivation, ability and importance to change and a trend toward significance for controlled motivation, *p* = 0.06). Patients reporting more psychological distress showed lower importance (*p* < 0.01) and confidence in their ability to change (*p* < 0.001) and lower autonomous motivation (*p* <. 05), whilst those with lower body mass index reported greater importance to change (*p* < 0.05). A trend toward significance indicated that greater work and social adjustment was associated with higher importance to change (*p* = 0.06). Higher controlled motivation and greater confidence in one’s own ability to change predicted lower drop-out from the 6 weeks assessment (*p* < 0.05). Finally, a trend toward significance (*p* = 0.06) was found for greater confidence in one’s own ability to change to predict lower drop-out from the treatment.

**TABLE 3:** Standardized coefficients of the structural equation model.

							Confidence in					
	Autonomous		Controlled		Importance		own ability		Drop-out from		Drop-out from	
	motivation		motivation		to change		to change		the assessment		the treatment	
						
	β	R^2^	β	R^2^	β	R^2^	β	R^2^	β	R^2^	β	R^2^
		0.293		0.148		0.281		0.448		0.273		0.199
Age	−0.15 (ns)		−0.01 (ns)		−0.17 (ns)		−0.08 (ns)		–		–	
Alliance with the therapist	0.39***		0.27 (*p* = 0.055)		0.28**		0.40***		–		–	
Work and Social Adjustment Scales	0.22 (ns)		0.24 (ns)		0.28 (*p* = 0.062)		0.08 (ns)		–		–	
Depression Anxiety and Stress Scales	−0.47*		−0.08 (ns)		−0.51**		−0.56***		–		–	
Cognitive style	0.04 (ns)		−0.07 (ns)		0.05 (ns)		0.08 (ns)		–		–	
Duration of illness	−0.18 (ns)		−0.08 (ns)		−0.01 (ns)		−0.16 (ns)		–		–	
Body Mass Index	−0.09 (ns)		−0.18 (ns)		−0.25*		−0.03 (ns)		–		–	
Eating Disorder Examination	0.21 (ns)		−0.03 (ns)		0.20 (ns)		0.11 (ns)		–		–	
Questionnaire												
Autonomous motivation	–		–		–		–		0.34 (ns)		0.19 (ns)	
Controlled motivation	–		–		–		–		−0.34*		−0.07 (ns)	
Importance to change	–		–		–		–		0.32 (ns)		0.36 (ns)	
Confidence in own ability to change	–		–		–		–		−0.46*		−0.46 (*p* = 0.056)	

Description of standardized coefficients of the variables included in the structural equation model. *p < 0.05; **p < 0.01; ***p < 0.001.

## Discussion

The aim of this study was to assess the feasibility of using a novel, online guided self-help program for patients with anorexia nervosa who had been assessed for outpatient treatment by examining drop-out rates. Rates of drop-out from the completion of the end-of-intervention assessment (end of intervention, at 6 weeks), rates of intervention adherence (defined as attendance of a minimum of four of six guided online sessions) and differences in baseline demographic (i.e., age, years of education) and clinical (i.e., illness severity) variables between drop-out and completers were explored. Differences between groups were also examined at the end of the first week of participation in the project, in relation to eating behavior, usage of self-help materials and satisfaction with the guidance provided. Finally, the relationship between motivation to change and drop-out was investigated. The hypotheses were that: (i) those who completed the end-of-intervention assessment or completed a minimum of four of six guided sessions would not be significantly different in demographic or clinical variables at baseline, compared to those who did not complete the assessment or the intervention, (ii) completers would show greater engagement with the guided self-help intervention and satisfaction with their mentor at the end of the first week, compared to non-completers, (iii) greater autonomous motivation to change at baseline would be associated with lower drop-out from the completion of the end-of-intervention measures and from the intervention.

Our results support the first hypothesis in that no baseline differences in socio-demographic or clinical variables were found between those who did and did not complete the 6-week assessment and between those who did and did not complete a minimum four guided sessions. This finding aligns with several studies examining drop-out from the use of technology-based interventions in patients with anorexia nervosa or bulimia nervosa (Schlegl et al., 2015). Results also support the second hypothesis, as patient- and treatment-related variables during the very first week of receiving the intervention differed between the completer and non-completer groups. Patients who dropped out (from assessment or the intervention) showed less satisfaction with their relationship with the mentor. Moreover, those who did not complete the end of intervention assessment showed a trend toward more frequent purging behaviors. These findings confirm the importance of considering patient- and early process-related variables when delivering technology-based interventions (Kelders et al., 2012) and are consistent with the literature indicating that low treatment credibility and poor early alliance with the therapist are associated with premature termination of treatment (Jordan et al., 2017). The poorer quality of the relationship with the mentor found in the group of non-completers is particularly important when considering the specific characteristics of *Recovery*MANTRA. The emphasis of the intervention is placed on increasing the patient’s confidence in their own ability to change by providing compassionate mentorship and promoting the use of the recovery narratives (i.e., video-clips) (Cardi et al., 2015). The poor agreement on the goals for the online sessions and the weak alliance with the mentor are likely to jeopardize the intervention’s outcomes. The greater frequency of purging behaviors at the end of the first week among those who did not complete the end-of-intervention measures also suggests that these behaviors might interfere with patients’ ability or willingness to adhere to the program. It is also possible that the materials offered were not specific enough to support patients with tackling these symptoms. Patients with anorexia nervosa presenting episodes of binging and purging have shown poorer emotion regulation skills when coping with negative emotions than patients presenting restrictive behaviors (Rowsell et al., 2016) and higher frequency of purging behaviors has been associated with worse treatment outcomes overall (Vall and Wade, 2015).

Current findings offer mixed results with regard to the third study hypothesis. As expected, patients reporting greater confidence in their own ability to change were less likely to drop-out from the assessment or the intervention. However, controlled motivation predicted drop-out from the assessment in an unexpected direction, with greater controlled motivation being associated with lower drop-out. This finding does not align with what has been previously found in the literature. Three studies in particular have investigated the role of autonomous and controlled motivation to change in patients with eating disorders (Mansour et al., 2012; Carter and Kelly, 2015; Thaler et al., 2016). These studies found that greater autonomous motivation for treatment predicted lower levels of eating disorder symptoms, or a faster improvement in these symptoms at the end of treatment (Mansour et al., 2012; Carter and Kelly, 2015; Thaler et al., 2016). The current work differs from those past studies in at least three ways: (i) it did assess drop-out, rather than treatment outcomes, (ii) it examined the predictive role of patient motivation over a shorter period of time and (iii) it examined a technology-based as opposed to standard face-to-face treatment for patients with anorexia nervosa. These differences might explain the divergence of the findings and also highlight that autonomous and controlled motivation to change are likely to have a complex role in treatment processes and outcomes for patients with anorexia nervosa, considering the high ambivalence toward change among this patient group (Schmidt and Treasure, 2006). Controlled motivation indicates an individual’s proneness to change due to expectations or pressure from others. Patients’ tendency to align with expectations from others at the beginning of treatment could have a protective role against non-adherence to treatment. This would validate models of treatment that encourage the involvement of close others in the care of adults with anorexia nervosa, such as the New Maudsley Approach (Treasure et al., 2016).

### Clinical Implications and Limitations

Sixty-seven out of 82 participants completed the 6 weeks assessment in this study, and they also attended a minimum of four out of six online guided sessions with a mentor. Across both groups (those who did and did not complete the 6 weeks assessment) 70 participants completed at least four guided sessions, of whom 59 participants completed all the six sessions offered. These rates of completion compare very favorably to the findings of a systematic review of 26 technology-based studies in eating disorders that reported mean compliance to treatment (defined as attendance to all treatment sessions) at 57.6% (ranging from 18.4 to 95.5%; Schlegl et al., 2015). Our rates also compare favorably to the finding that 20–40% for patients with anorexia nervosa do not complete standard, psychotherapy-based interventions (DeJong et al., 2012). Based on this evidence, it seems plausible to state that technology-based guided self-help for anorexia nervosa is acceptable and is not associated with lower adherence than standard treatment. A recent study also found that an online, guided self-help intervention designed to prevent relapse from intensive treatment was beneficial in the aftercare of inpatients with anorexia nervosa (Schmidt et al., 2017). However, these findings cannot generalize to the use of standalone online interventions to replace standard treatment or as only form of support after care in anorexia nervosa.

The finding that non-completers report lower satisfaction with their online mentor after the first week of participation in the program highlights the importance of attending early to the quality of the working alliance and the need to ensure that the work of the mentors is closely and regularly monitored, especially when guidance is delivered by non-professionals. Our research group supervised mentors once a week and trained them in the use of motivational interviewing techniques (Cardi et al., 2015). A greater emphasis on early fidelity to the intervention and use of the self-help materials might improve overall satisfaction with the mentorship among those who (are likely to) drop-out from the intervention. In our study, we contacted participants who were not completing the online sessions and assessments a maximum of four times (once/week for 3 weeks and once more after 20 days). Those who dropped out soon after the completion of the baseline questionnaires and never started the online sessions did not reply to any of our emails. Those who dropped-out after the first or first two online sessions and who also provided feedback to us expressed worries about confidentiality (*n* = 1), difficulties due to work commitments (*n* = 1), perceived lack of availability of the mentor (*n* = 1), a preference for face-to-face therapy (*n* = 1), and being too ill (*n* = 1) to continue with the project. This suggests that treatment preferences, beliefs about the illness and difficulties with synchronous guidance play a role in early drop-out from online interventions. Type and extent of previous treatments could also predict early drop-out from these interventions.

## Conclusion

To conclude, the findings of this study indicate that online guided self-help offered to patients with anorexia nervosa who have been assessed to receive outpatient treatment is acceptable and feasible. To a certain degree, a patient’s tendency to adhere to treatment because of external pressure or expectations from others seems to play a protective role in completing the online intervention. More work is needed on monitoring patients’ clinical symptoms and expectations and satisfaction with the program during the earlier phases of their participation to reduce the risk of drop-out.

## Data Availability Statement

The datasets generated for this study are available on request to the corresponding author.

## Ethics Statement

The study involved human participants and was reviewed and approved by the Research Ethics Committee of London-Brent, project reference number: 14-LO-1347. The patients/participants provided their written informed consent to participate in this study.

## Author Contributions

VC and JT contributed to study’s conceptualization, investigation, data curation, funding acquisition, project administration, and writing up of the manuscript. GA contributed to project admin, investigation, data curation, formal analysis, and writing up of the manuscript. LS and GL contributed to conceptualization, data curation, formal analysis, and writing up of the manuscript. SA, US, and PM contributed to conceptualization, investigation, funding acquisition, and writing up of the manuscript.

## Conflict of Interest

The authors declare that the research was conducted in the absence of any commercial or financial relationships that could be construed as a potential conflict of interest.
