# Supplementary material for: The Feasibility of Using Guided Self-Help in Anorexia Nervosa: An Analysis of Drop-Out From the Study Protocol and Intervention Adherence
Source: Front Psychol. 2020 Apr 16;11:707. doi: 10.3389/fpsyg.2020.00707 (PMC7178255; doi:10.3389/fpsyg.2020.00707)
Supplement: Supplementary file 1 [file Data_Sheet_1.docx]

**Supplementary Materials**

*Motivational Ruler*

***INSTRUCTIONS:*** Using the ruler above, please circle the number that best describes how you CURRENTLY think about your ED:

**Importance to change. Please ask yourself the following questions:**

**How important is for you to change? What score would you give yourself out of 10?**

(Not at all) 1 2 3 4 5 6 7 8 9 10 (Extremely important)

**Ability to change. Please ask yourself the following questions:**

**How confident are you in your ability to change? What score would you give yourself out of 10?**

(Not at all) 1 2 3 4 5 6 7 8 9 10 (Extremely confident)

*Cognitive and Behavioral flexibility*

Please indicate your CURRENT thoughts and behaviors using the following scale:

1. How often do your thoughts involve paying very strict attention to small details?

1. How often do your thoughts involve focusing on the “big picture”?

1. How often do you find yourself closely sticking to rules and rituals in your behaviour?

1. How often do you feel comfortable behaving in a spontaneous manner?

*Alliance with therapist*

Please indicate your responses using the following scale:

1. How often do you feel like your therapist understands you?

1. How often do you feel confident that your therapist is pointing you in the right direction?

1. How often do you feel that you and your therapist are working toward mutually agreed upon goals?

1. How often do you trust your therapist?

1. How often do you feel that your therapist is offering you new ways of looking at your problem?
